# Allogeneic stem cell transplant recipients admitted to the intensive care unit during the peri-transplant period have unfavorable outcomes—results of a retrospective analysis from a German university hospital

**DOI:** 10.1007/s00277-021-04698-3

**Published:** 2021-10-20

**Authors:** Jorge Garcia Borrega, Jan-Michel Heger, Philipp Koehler, Udo Holtick, Michael Hallek, Christof Scheid, Boris Böll, Alexander Shimabukuro-Vornhagen, Matthias Kochanek, Dennis A. Eichenauer

**Affiliations:** 1 First Department of Internal Medicine, Center for Integrated Oncology Aachen Bonn Cologne Dusseldorf, University of Cologne, Cologne, Germany; 2 Faculty of Medicine and University Hospital Cologne, Cologne Excellence Cluster On Cellular Stress Responses in Aging-Associated Diseases (CECAD), University of Cologne, , Cologne, Germany; 3 First Department of Internal Medicine, University Hospital Cologne, Kerpener Str. 62, D-50937 Cologne, Germany

**Keywords:** Allogeneic stem cell transplantation, Intensive care unit, Mechanical ventilation, Renal replacement therapy, Prognosis

## Abstract

The prognosis of allogeneic stem cell transplant recipients admitted to the intensive care unit (ICU) has improved over the last decades. However, data focusing on patients treated in the ICU during the peri-transplant period are scarce. We therefore conducted an analysis comprising 70 patients who had allogeneic stem cell transplantation at the University Hospital Cologne between 2014 and 2020 and were admitted to the ICU between the initiation of conditioning therapy and day 30 after transplantation. The median age was 59 years (range: 18 − 72 years). 50% of patients were female. Sepsis was the most common cause for ICU admission (49%). Mechanical ventilation (MV) was required in 56% of patients, 27% had renal replacement therapy (RRT), and 64% needed vasopressors. The ICU, hospital, 90-day, and 1-year survival rates were 48.6%, 38.6%, 35.7%, and 16.2%, respectively. MV and/or RRT during the ICU stay were associated with an impaired survival (*p* < 0.0001). The same was true for the use of vasopressors (*p* < 0.0001). In contrast, baseline characteristics did not impact the outcome. Cardiopulmonary resuscitation (CPR) was performed in 17% of patients. None of the patients undergoing CPR was alive at 1 year. Among patients who died after discharge from the ICU (*n* = 23), sepsis and other infectious complications represented the major causes of death (48%). Taken together, the present analysis indicates unfavorable outcomes for allogeneic stem cell transplant recipients admitted to the ICU during the peri-transplant period. The data may help to make informed decisions with patients and their families.

**Supplementary Information:**

The online version contains supplementary material available at 10.1007/s00277-021-04698-3.

## Introduction

The prognosis of allogeneic stem cell transplant recipients admitted to the intensive care unit (ICU) has improved over the last decades [1, 2]. A large retrospective study analyzed the outcome of 330 patients who had undergone allogeneic stem cell transplantation (aSCT) between 2000 and 2013 and had been admitted to the ICU at least once thereafter. The ICU and hospital survival rates improved from 44 and 26%, respectively, for patients treated on the ICU between 2000 and 2006 to 60% and 43%, respectively, for patients who had treatment on the ICU between 2007 and 2013. However, several factors remain associated with a poor prognosis for critically ill allogeneic stem cell transplant recipients. Those include mechanical ventilation (MV), renal replacement therapy (RRT), the use of vasopressors, liver impairment, and graft-versus-host disease (GvHD) [1, 3–5].

Reports focusing on patients admitted to the ICU during the peri-transplant period are scarce [6, 7]. To shed more light on characteristics and course of this patient group, we conducted an analysis including allogeneic stem cell transplant recipients who required treatment in the ICU between the initiation of conditioning therapy and day 30 after transplantation.

## Patients and methods

Patients aged ≥ 18 years who had aSCT at the University Hospital Cologne between January 1, 2014, and December 31, 2020, and were admitted to the ICU during the peri-transplant period (defined as the time between the initiation of conditioning therapy and day 30 after transplantation) were included in the present analysis. Patient characteristics, laboratory parameters, the Hematopoietic Cell Transplantation-specific Comorbidity Index (HCT-CI) score (a score consisting of comorbidities and predicting non-relapse mortality and survival in patients undergoing aSCT) at initiation of conditioning therapy, aSCT-related information, causes for ICU admission, the Sequential Organ Failure Assessment (SOFA) score (a score describing organ function and extent of organ failure in critically ill patients) at ICU admission, and procedures performed during the stay on the ICU were extracted from the patient charts [8, 9].

Numbers and proportions were indicated for categorical variables. Medians and ranges were calculated for continuous variables. Survival curves were obtained using the Kaplan–Meier method. Overall survival (OS) was defined as the time from admission to the ICU until death and was censored at the time of last information for surviving patients. The influence of variables on OS was investigated using the log-rank test (Mantel-Cox). Statistical significance was set to *p* < 0.05 (two-sided). The statistical analyses were performed using Microsoft Excel (version 16.45), SPSS (IBM, version 27.0.1.0), and R-project/RStudio software (version 3.6.2 /1.4.1103) for Mac as well as GraphPad Prism (version 8.0.1) for Windows.

## Results

### Baseline patient characteristics

Between January 1, 2014, and December 31, 2020, 638 patients had aSCT at the University Hospital Cologne. Of these, 70 (11.0%) were admitted to the ICU between the initiation of conditioning therapy and day 30 after transplantation. Among the patients aged younger than 40 years at the time of aSCT, ICU admission during the peri-transplant period was necessary in 4.8% of cases. In contrast, patients aged 40 to 60 years and older than 60 years were admitted to the ICU in 14.1% and 11.3% of cases, respectively (data not shown). Patients necessitating treatment on the ICU had a median age of 59 years (range: 18–72 years) at aSCT. Males and females accounted for 35/70 patients (50%) each. Acute leukemia and myelodysplastic syndrome represented the most common indications for aSCT (54/70 patients; 77.1%). Less frequent indications were non-Hodgkin lymphomas (10/70 patients; 14.3%) and myeloproliferative neoplasms (4/70 patients; 5.7%). The median HCT-CI score at the initiation of conditioning therapy was 4 (range: 0–10). Matched unrelated donors represented the most common stem cell source (39/70 patients; 55.7%) (Table 1).

**Table 1:** Patient characteristics of patients admitted to the ICU between the initiation of conditioning therapy and day 30 after aSCT

		**%**
Total patients (*n*)	70	
Age—median (range)	59 (18–72)	
Female sex (*n*)	35	50
Indication for aSCT		
Acute leukemia or MDS (*n*)	54	77.1
NHL (*n*)	10	14.3
MPN (*n*)	4	5.7
Other (*n*)	2	2.9
Remission status prior to aSCT		
CR (*n*)	24	34.3
PR (*n*)	19	27.1
SD (*n*)	4	5.7
MRD positive (*n*)	7	10.0
PD (*n*)	16	22.9
HCT-CI score median (range)	4 (0–10)	
Type of donor		
Haplo (*n*)	9	12.9
MMUD (*n*)	15	21.4
MUD (*n*)	39	55.7
SIB (*n*)	7	10.0
Acute GvHD		
Grade 1/2 (n)	26	37.1
Grade 3/4 (n)	10	14.3

*aSCT*, allogeneic stem cell transplantation; *MDS*, myelodysplastic syndrome; *NHL*, non-Hodgkin lymphoma; *MPN*, myeloproliferative neoplasm; *CR*, complete remission; *PR*, partial remission; *SD*, stable disease; *MRD*, measurable residual disease; *PD*, progressive disease; *HCT-CI*, Hematopoietic Cell Transplantation-specific Comorbidity Index; *MMUD*, mismatched unrelated donor; *MUD*, matched unrelated donor; *SIB*, matched-related sibling; *GvHD*, graft-versus-host disease

### Characteristics of ICU admission and procedures on the ICU

The median time interval between aSCT and admission to the ICU was 6.5 days (range: day −12–day 29). ICU admission occurred during conditioning therapy in 22/70 patients (31.4%) whereas 48/70 patients (68.6%) were admitted after aSCT (Table 2, supplemental Fig. 1).

**Table 2:** ICU characteristics of patients admitted to the ICU between the initiation of conditioning therapy and day 30 after aSCT

		**%**
Admission (days from aSCT)—median (range)	6.5 (− 12–29)	
Leading cause for admission		
Arrhythmia (*n*)	5	7.1
Bleeding (*n*)	1	1.4
IHCA (*n*)	2	2.9
STEMI (*n*)	1	1.4
Neurological symptoms (*n*)	13	18.6
Post-surgery surveillance (*n*)	1	1.4
Respiratory failure (*n*)	13	18.6
Sepsis (*n*)	34	48.6
Duration of ICU stay (days)—median (range)	5 (1–42)	
MV (*n*)	39	55.7
Days from admission to MV—median (range)	1.5 (1–55)	
Duration of MV (days)—median (range)	3.5 (1–25)	
NIV (*n*)	2	2.9
High-flow nasal cannula (*n*)	4	5.7
RRT (*n*)	19	27.1
Vasopressor (*n*)	45	64.3
CPR (*n*)	12	17.1
SOFA score at admission—median (range)	9.5 (1–21)	
Lactate [mmol/l] at admission—median (range)	1.6 (0.5–19)	

*aSCT*, allogeneic stem cell transplantation; *IHCA*, in-hospital cardiac arrest; *STEMI*, ST-elevation myocardial infarction; *ICU*, intensive care unit; *MV*, mechanical ventilation; *NIV*, non-invasive ventilation; *RRT*, renal replacement therapy; *CPR*, cardiopulmonary resuscitation; *SOFA*, Sequential Organ Failure Assessment

The most frequent causes for ICU admission were sepsis (34/70 patients; 48.6%), respiratory failure (13/70 patients; 18.6%), and neurological symptoms (13/70 patients; 18.6%). In patients presenting with fever, diagnostic workup including the collection of blood cultures had already been conducted and treatment with broad-spectrum antibiotics (piperacillin/tazobactam or meropenem in most cases) had already been initiated before ICU admission. The median SOFA score at the time of ICU admission was 9.5 (range: 1–21). 39/70 patients (55.7%) had MV (including one patient who had veno-venous extracorporeal membrane oxygenation), 19/70 patients (27.1%) underwent RRT and 45/70 patients (64.3%) required vasopressors during the ICU stay. Cardiopulmonary resuscitation (CPR) was performed in 12/70 patients (17.1%). The median duration of stay on the ICU was 5 days (range: 1–42 days) (Table 2).

### Outcome and risk factors

The median observation time was 45.5 days (range: 1–2266 days) for all patients and 881 days (range: 151–2266 days) for surviving patients. The ICU, hospital, 90-day, and 1-year survival rates were 48.6%, 38.6%, 35.7%, and 16.2%, respectively (Table 3). In contrast, the 1-year survival rate for allogeneic stem cell transplant recipients who did not require treatment in the ICU during the peri-transplant period (data available for 472/568 patients; 83.1%) was 77.6% (data not shown).

**Table 3:** Outcome characteristics of patients admitted to the ICU between initiation of conditioning therapy and day 30 after aSCT

		**%**
ICU survival (*n*)	34/70	48.6
Hospital survival (*n*)	27/70	38.6
90-day survival (*n*)	25/70	35.7
1-year survival (*n*)	11/68	16.2
Follow-up (days)—median (range) (all patients)	45.5 (1–2266)	
Follow-up (days)—median (range) (survivors)	881 (151–2266)	
Time from ICU admission to death (days)—median (range)	12 (1–1228)	
Time from aSCT to death (days)—median (range)	27 (1–1238)	
Cause of death among ICU survivors		
Sepsis (*n*)	7	30.4
Underlying malignancy (*n*)	4	17.4
Unknown (*n*)	3	13.0
GvHD (*n*)	3	13.0
Other infection (*n*)	4	17.4
Cardiovascular disease (*n*)	2	8.7

*ICU*, intensive care unit; *aSCT*, allogeneic stem cell transplantation; *GvHD*, graft-versus-host disease

**Fig. 1:**
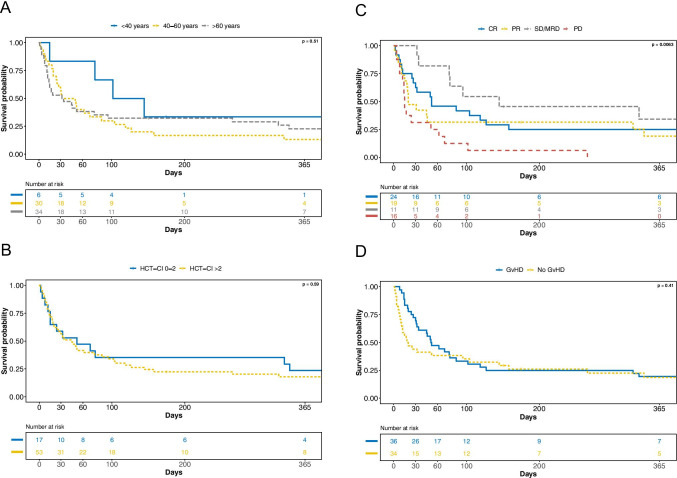
**A** Overall survival according to age (< 40 years vs 40–60 years vs > 60 years). **B** Overall survival according to HCT-CI (0–2 vs > 2). **C** Overall survival according to remission status prior to aSCT (CR vs PR vs SD/MRD positive vs PD). **D** Overall survival according to the presence of acute GvHD. Legend: HCT-CI, Hematopoietic Cell Transplantation-specific Comorbidity Index; aSCT, allogeneic stem cell transplantation; CR, complete remission; PR, partial remission; SD, stable disease; MRD, measurable residual disease; PD, progressive disease; GvHD, graft-versus-host disease

**Fig. 2:**
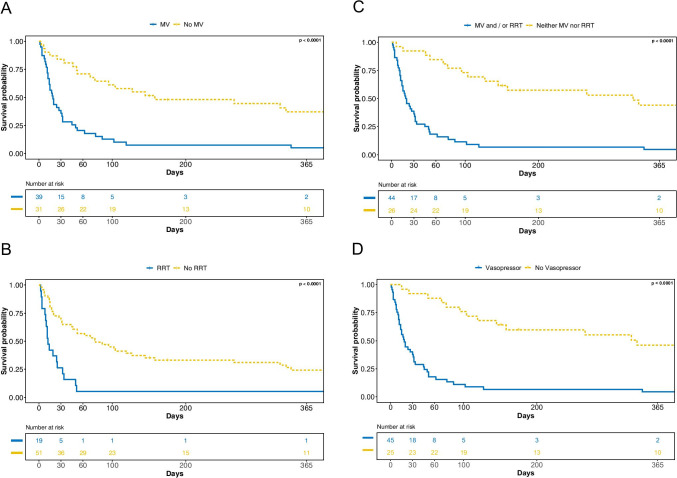
**A** Overall survival according to the necessity of MV. **B** Overall survival according to the necessity of RRT. **C** Overall survival according to the necessity of MV and/or RRT (MV and/or RRT vs no MV and/or RRT). **D** Overall survival according to the necessity to use vasopressors. Legend: MV, mechanical ventilation; RRT, renal replacement therapy

The median time interval between ICU admission and death was 12 days (range: 1–1228 days). The median time from aSCT to death was 27 days (range 1–1238 days). The most frequent causes of death among the 23 patients who were discharged from the ICU but died during observation were sepsis (7/23 patients; 30.4%), other infectious complications (4/23 patients; 17.4%), relapse or progression of the underlying malignancy (4/23 patients; 17.4%) and GvHD (3/23 patients; 13.0%). Neither age (*p* = 0.51) and HCT-CI score (*p* = 0.59) nor the presence of GvHD during the stay on the ICU (*p* = 0.41) had an impact on the OS (Fig. 1A, B, D; supplemental Table 1). In contrast, progression of the underlying malignancy at the initiation of conditioning therapy (*p* = 0.0063), MV (*p* < 0.0001) and/or RRT (*p* < 0.0001) and use of vasopressors (*p* < 0.0001) during the ICU stay were associated with an impaired OS (Figs. 1C and  2). None of the 16 patients who had progression of the underlying malignancy at the initiation of conditioning therapy survived 1 year. Only 2/39 patients (5.1%) who had required MV, 1/19 patients (5.3%) who had undergone RRT, and 2/45 patients (4.4%) necessitating vasopressors were alive at 1 year. There were no survivors among the patients in which CPR was performed.

## Discussion

Data on characteristics and course of allogeneic stem cell transplant recipients admitted to the ICU during the peri-transplant period are scarce. We therefore performed a single-center retrospective analysis comprising 70 patients treated on the ICU between the initiation of conditioning therapy and day 30 after transplantation. The major findings were as follows: 1) 11.0% of allogeneic stem cell transplant recipients required treatment on the ICU during the peri-transplant period; 2) Despite an ICU survival rate close to 50%, the 1-year OS of patients treated on the ICU during the peri-transplant period was only 16.2%; 3) Only 2/44 patients (4.5%) requiring MV and/or RRT, 2/45 patients (4.4%) necessitating vasopressors, and no patient undergoing CPR were alive at 1 year.

In the present analysis, 11.0% of allogeneic stem cell transplant recipients were admitted to the ICU between the initiation of conditioning therapy and day 30 after transplantation. Patients had a median age of 59 years. Males and females accounted for 50% of cases each. Hence, the ICU admission rate was comparable to previous studies from Germany and the US (ICU admission rates: 14.9% and 13.0%, respectively) including patients that had been hospitalized for aSCT. The median age and the proportion of females in the present analysis were slightly higher than in the previous reports (median age: 54.4 years and 52 years, respectively; proportion of females: 42.3% and 42%, respectively) [6, 7].

The most common cause for ICU admission in the present analysis was sepsis (34/70 patients; 48.6%). The median SOFA score at the time of ICU admission was 9.5 and thus lower than in the already mentioned German study that had reported a median SOFA score of 14 [7]. However, the lower median SOFA score did not result in improved ICU and 1-year survival rates. This is in contrast to earlier studies [10, 11]. For instance, a retrospective Swedish analysis evaluating the course of critically ill allogeneic stem cell transplant recipients was able to discriminate 3 risk groups according to the SOFA score at ICU admission (risk group 1: SOFA score < 8; risk group 2: SOFA score 8–11; risk group 3: SOFA score > 11) [10].

The present analysis indicated a dismal prognosis for the 16 patients who presented with progression of the underlying malignancy at the initiation of conditioning therapy. None of these patients was alive at 1 year. This finding is consistent with studies evaluating the impact of the remission status on the outcome of allogeneic stem cell transplant recipients. A recent analysis comprising 392 patients who had reduced-intensity or non-myeloablative aSCT for acute myeloid leukemia revealed inferior event-free survival and overall survival rates for patients with active disease prior to aSCT (*n* = 130) as compared with patients who had measurable residual disease (MRD) but no increased blast count (*n* = 115) and individuals with no MRD (*n* = 147), respectively [12].

Overall, 39/70 patients (55.7%) taken into account for the present analysis required MV and 19/70 patients (27.1%) had RRT. Thus, the proportion of individuals who had MV and/or RRT was similar to previous studies including patients hospitalized for aSCT [6, 7]. In the present analysis, only 2 patients necessitating MV and 1 patient requiring RRT were alive at 1 year. This is also in agreement with previous publications consistently reporting poor outcomes for critically ill allogeneic stem cell transplant recipients undergoing MV and/or RRT [6, 7, 13, 14]. Death rates for patients who had RRT were up to 100% [7, 15].

The ICU, hospital, 90-day, and 1-year survival rates for the 70 patients included in the present analysis were 48.6%, 38.6%, 35.7%, and 16.2%, respectively. A previous analysis evaluating characteristics and outcomes of 78 patients admitted to the ICU during hospitalization for aSCT indicated similar results (ICU survival: 56.4%; 100-day survival: 42.3%) [7]. In contrast, analyses investigating critically ill allogeneic stem cell transplant recipients irrespective of the time interval between aSCT and ICU admission reported better survival outcomes. According to two recent studies, almost 50% of patients survived 90 days and roughly 30% were alive at 1 year [1, 2].

Besides its retrospective single-center design, the present analysis has some limitations. Those include the inability to calculate the Prognostic Index For Intensive Care After Allogeneic Stem Cell Transplantation (PICAT) due to insufficient information regarding some parameters contained in this score that allows the allocation of critically ill allogeneic stem cell transplant recipients into three distinct risk groups with hospital mortality rates ranging between 34 and 91% [16].

Taken together, the present study confirms that patients admitted to the ICU during the peri-transplant period have unfavorable outcomes. Admission to the ICU is nonetheless justified given the long-term survival of a significant minority of patients. However, in line with previous reports, the importance of advance care planning in allogeneic stem cell transplant recipients is underscored given the high mortality especially in individuals developing multi-organ failure [17–19]. A time-limited trial of intensive care treatment for 3 to 5 days can represent an option in this patient group. If the patient´s condition improves during the determined time interval, intensive care treatment is being continued whereas treatment goals are shifted towards palliation and reduction of the symptom burden alone if the condition deteriorates [20].

## Supplementary Information

Below is the link to the electronic supplementary material.

Supplementary Table 1 (DOCX 27 KB)

Supplementary Fig. 1 Distribution of ICU admission times (counted from the day of aSCT). Legend: ICU: intensive care unit. (PDF 11 KB)

## Data Availability

The data generated and analyzed are available upon request. Decisions in terms of data sharing will be made on a case-by-case basis.

## References

[1] Lueck C, Stadler M, Koenecke C, Hoeper MM, Dammann E, Schneider A, Kielstein JT, Ganser A, Eder M, Beutel G (2018) Improved short- and long-term outcome of allogeneic stem cell recipients admitted to the intensive care unit: a retrospective longitudinal analysis of 942 patients. Intensive Care Med 44.10.1007/s00134-018-5347-x 10.1007/s00134-018-5347-x 30141173

[2] Lengliné E, Chevret S, Moreau A-S, Pène F, Blot F, Bourhis J-H, Buzyn A, Schlemmer B, Socié G, Azoulay E (2015) Changes in intensive care for allogeneic hematopoietic stem cell transplant recipients. Bone Marrow Transplant 50.10.1038/bmt.2015.55 10.1038/bmt.2015.55 25798675

[3] Saillard C, Darmon M, Bisbal M, Sannini A, Chow-Chine L, Faucher M, Lengline E, Vey N, Blaise D, Azoulay E, Mokart D (2018). Critically ill allogenic HSCT patients in the intensive care unit: a systematic review and meta-analysis of prognostic factors of mortality. Bone Marrow Transplant.

[4] Orvain C, Beloncle F, Hamel J-F, Thépot S, Mercier M, Kouatchet A, Farhi J, Francois S, Guardiola P, Asfar P, Hunault-Berger M, Mercat A, Ifrah N, Tanguy-Schmidt A (2017) Different impact of the number of organ failures and graft-versus-host disease on the outcome of allogeneic stem cell transplantation recipients requiring intensive care. Transplantation 101.10.1097/TP.0000000000001143 10.1097/TP.0000000000001143 26950729

[5] Moreau A-S, Seguin A, Lemiale V, Yakoub-Agha I, Girardie P, Robriquet L, Mangalaboyi J, Fourrier F, Jourdain M (2014) Survival and prognostic factors of allogeneic hematopoietic stem cell transplant recipients admitted to intensive care unit. Leuk Lymphoma 55.10.3109/10428194.2013.836602 10.3109/10428194.2013.836602 23964648

[6] Mayer S, Pastores SM, Riedel E, Maloy M, Jakubowski AA (2017) Short- and long-term outcomes of adult allogeneic hematopoietic stem cell transplant patients admitted to the intensive care unit in the peritransplant period. Leuk Lymphoma 58.10.1080/10428194.2016.1195499 10.1080/10428194.2016.1195499 PMC5581948 27347608

[7] Michel CS, Teschner D, Schmidtmann I, Theobald M, Hauptrock B, Wagner-Drouet EM, Radsak MP (2019). Prognostic factors and outcome of adult allogeneic hematopoietic stem cell transplantation patients admitted to intensive care unit during transplant hospitalization. Sci Rep.

[8] Sorror ML, Maris MB, Storb R, Baron F, Sandmaier BM, Maloney DG, Storer B (2005) Hematopoietic cell transplantation (HCT)-specific comorbidity index: a new tool for risk assessment before allogeneic HCT. Blood 106.10.1182/blood-2005-05-2004 10.1182/blood-2005-05-2004 PMC1895304 15994282

[9] Vincent J-L, Moreno R, Takala J, Willatts S, De Mendonça A, Bruining H, Reinhart CK, Suter PM, Thijs LG (1996) The SOFA (Sepsis-related Organ Failure Assessment) score to describe organ dysfunction/failure. Intensive Care Med 22.10.1007/BF01709751 10.1007/BF01709751 8844239

[10] Gilli K, Remberger M, Hjelmqvist H, Ringden O, Mattsson J (2010) Sequential Organ Failure Assessment predicts the outcome of SCT recipients admitted to intensive care unit. Bone Marrow Transplant 45.10.1038/bmt.2009.220 10.1038/bmt.2009.220 19718056

[11] Platon L, Amigues L, Ceballos P, Fegueux N, Daubin D, Besnard N, Larcher R, Landreau L, Agostini C, Machado S, Jonquet O, Klouche K (2016). A reappraisal of ICU and long-term outcome of allogeneic hematopoietic stem cell transplantation patients and reassessment of prognosis factors: results of a 5-year cohort study (2009–2013). Bone Marrow Transplant.

[12] Jentzsch M, Grimm J, Bill M, Brauer D, Backhaus D, Schulz J, Goldmann K, Niederwieser D, Platzbecker U, Schwind S (2021) Prognostic relevance of remission and measurable residual disease status in AML patients prior to reduced intensity or non-myeloablative allogeneic stem cell transplantation. Blood Cancer J 11.10.1038/s41408-021-00471-x 10.1038/s41408-021-00471-x PMC8084997 33927190

[13] Hamidi M, Gossack-Keenan KL, Ferreyro BL, Angriman F, Rochwerg B, Mehta S (2019) Outcomes of hematopoietic cell transplant recipients requiring invasive mechanical ventilation: a two-centre retrospective cohort study. Can J Anesth Can d’anesthésie 66.10.1007/s12630-019-01439-z 10.1007/s12630-019-01439-z 31290122

[14] Lindgaard SC, Nielsen J, Lindmark A, Sengeløv H (2016). Prognosis of Allogeneic haematopoietic stem cell recipients admitted to the intensive care unit: a retrospective, single-centre study. Acta Haematol.

[15] Gilbert C, Vasu TS, Baram M (2013). Use of mechanical ventilation and renal replacement therapy in critically ill hematopoietic stem cell transplant recipients. Biol Blood Marrow Transplant.

[16] Bayraktar UD, Milton DR, Shpall EJ, Rondon G, Price KJ, Champlin RE, Nates JL (2017) Prognostic index for critically ill allogeneic transplantation patients. Biol Blood Marrow Transplant 23.10.1016/j.bbmt.2017.03.003 10.1016/j.bbmt.2017.03.003 28263919

[17] Wang WS, Ma JD, Nelson SH, Revta C, Buckholz GT, Mulroney CM, Roeland EJ (2017) Advance care planning and palliative care integration for patients undergoing hematopoietic stem-cell transplantation. J Oncol Pract 13.10.1200/JOP.2016.020321 10.1200/JOP.2016.020321 PMC6366810 28644706

[18] Loggers ET, LeBlanc TW, El-Jawahri A, Fihn J, Bumpus M, David J, Horak P, Lee SJ (2016). Pretransplantation supportive and palliative care consultation for high-risk hematopoietic cell transplantation patients. Biol Blood Marrow Transplant.

[19] Johnston EE, Muffly L, Alvarez E, Saynina O, Sanders LM, Bhatia S, Chamberlain LJ (2018) End-of-life care intensity in patients undergoing allogeneic hematopoietic cell transplantation: a population-level analysis. J Clin Oncol 36.10.1200/JCO.2018.78.0957 10.1200/JCO.2018.78.0957 PMC6324087 30183467

[20] Vink EE, Azoulay E, Caplan A, Kompanje EJO, Bakker J (2018) Time-limited trial of intensive care treatment: an overview of current literature. Intensive Care Med 44.10.1007/s00134-018-5339-x 10.1007/s00134-018-5339-x 30136140

