# Supplementary material for: Allogeneic stem cell transplant recipients admitted to the intensive care unit during the peri-transplant period have unfavorable outcomes—results of a retrospective analysis from a German university hospital
Source: Ann Hematol. 2021 Oct 20;101(2):389–95. doi: 10.1007/s00277-021-04698-3 (PMC8742807; doi:10.1007/s00277-021-04698-3)
Supplement: Supplementary file 1 — Supplementary Table 1 (DOCX 27 KB) [file 277_2021_4698_MOESM1_ESM.docx]

**Supplemental Table 1**

Comparison of age, HCT-CI and engraftment (defined as 500 neutrophils/µl at the time of ICU admission) between ICU survivors and non-survivors.

Legend: HCT-CI: Hematopoietic Cell Transplantation-specific Comorbidity Index; ICU: intensive care unit

| **Supplemental table 1** |  |  |
| --- | --- | --- |
|  | **Survivors (n=11)** | **Non-survivors (n=59)** |
| **Age - median (range)** | 59 (18 - 71) | 59 (25 - 72) |
| **HCT-CI score median (range)** | 4 (0 - 7) | 4 (0 - 10) |
| **Engrafted - n (%)** | 3/11 (27.3) | 17/59 (28.8) |
