# Supplementary figures and images for: Allogeneic stem cell transplant recipients admitted to the intensive care unit during the peri-transplant period have unfavorable outcomes—results of a retrospective analysis from a German university hospital
Source: Ann Hematol. 2021 Oct 20;101(2):389–95. doi: 10.1007/s00277-021-04698-3 (PMC8742807; doi:10.1007/s00277-021-04698-3)

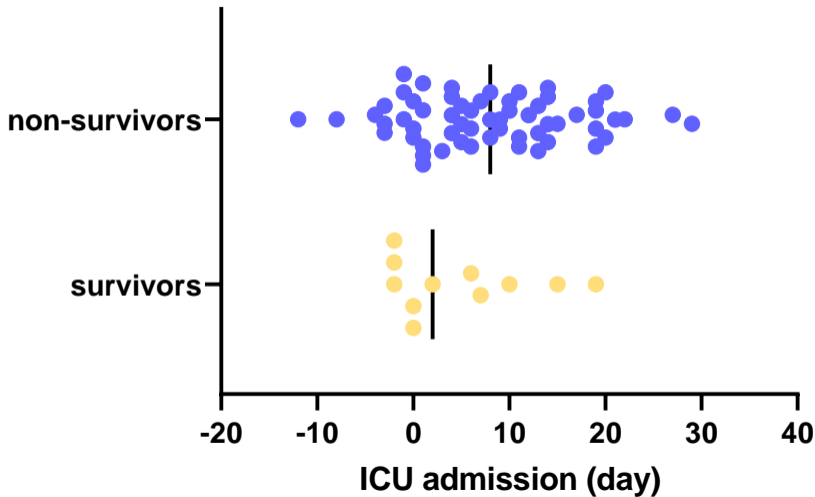

Supplement: Supplementary file 2 — Supplementary Fig. 1 Distribution of ICU admission times (counted from the day of aSCT). Legend: ICU: intensive care unit. (PDF 11 KB) [file 277_2021_4698_MOESM2_ESM.pdf]
